# Carnitine deficiency presenting with a decreased mental state in a patient with amyotrophic lateral sclerosis receiving long-term tube feeding: a case report

**DOI:** 10.1186/1752-1947-7-286

**Published:** 2013-12-30

**Authors:** Naohi Isse, Yoh Miura, Toshiyuki Obata, Noriko Takahara

**Affiliations:** 1 Department of Internal Medicine, Ako City Hospital, 1090 Nakahiro, Ako 678-0232, Hyogo, Japan; 2 Miura Clinic, 173-14 Daimochi, Kamigoori-cho, Ako-gun, Hyogo 678-1233, Japan

**Keywords:** Amyotrophic lateral sclerosis, Carnitine deficiency, Communication, Mental state, Tube feeding

## Abstract

**Introduction:**

L-carnitine is an important metabolic mediator involved in fatty acid transport. It is obtained from the diet, particularly from animal products, such as red meat. Previous reports have revealed that long-term tube feeding with a commercial product containing no or low levels of carnitine can lead to an altered mental state caused by hyperammonemia.

**Case presentation:**

A 72-year-old Japanese man had a 12-year history of amyotrophic lateral sclerosis. He was bedridden and had required mechanical ventilation and enteral tube feeding for 10 years at home. His main enteral solution was a commercial product that contained low carnitine levels, and he sometimes received coffee and homemade products such as miso soup. Our patient’s ability to communicate gradually deteriorated over a period of one year. His serum total carnitine level was abnormally low, at 26.7μmol/L (normal range, 45 to 91μmol/L), but his ammonium level was normal. His mental state improved dramatically after starting L-carnitine supplementation (600mg twice daily).

**Conclusion:**

This case highlights the importance of avoiding carnitine deficiency in patients with amyotrophic lateral sclerosis undergoing long-term tube feeding. These patients experience progressive muscle atrophy that might cause impaired carnitine storage and might manifest as communication difficulties. Carnitine deficiency can be misdiagnosed as a progression of systemic muscle atrophy. Clinicians should be aware of this disorder and should consider periodically measuring carnitine levels, regardless of the patient’s serum ammonium levels.

## Introduction

L-carnitine is a natural constituent of higher organisms, including animal cells [1]. It is mainly obtained from dietary sources, particularly red meat and dairy products. Previous reports have revealed that long-term tube feeding with commercial products containing low or no carnitine can contribute to altered mental states caused by hyperammonemia [2]. Lack of carnitine causes the accumulation of unoxidized fatty acids, which inhibit the urea cycle, resulting in hyperammonemia. Adults with chronic illnesses such as advanced cancer and chronic renal failure with hemodialysis are prone to carnitine deficiency because of decreased intake, increased utilization, or increased elimination of carnitine. Intravenous L-carnitine treatment improved patient-assessed fatigue in patients undergoing hemodialysis [3]. However, L-carnitine supplementation to treat carnitine deficiency did not improve fatigue in patients with invasive malignancies and good performance status [4].

Amyotrophic lateral sclerosis (ALS) is a progressive neurodegenerative disease that results in severe muscle atrophy. There is no effective treatment, and patients with ALS usually require mechanical ventilation and long-term tube feeding. We describe the case of a patient with ALS who required long-term tube feeding and whose mental state deteriorated because of carnitine deficiency, despite normal ammonium levels.

## Case presentation

A 72-year-old Japanese man had a 12-year history of ALS. He was bedridden and had been on mechanical ventilation and percutaneous endoscopic gastrostomy (PEG) tube feeding at home for the previous 10 years. He was fed 1000mL (1000kcal) of a commercial product (Ensure liquid®; Abbott Japan, Tokyo, Japan) containing <1nmol/mL of carnitine per day. He was also sometimes given homemade products such as miso soup and coffee with milk, all via the PEG tube. Over one year, his ability to communicate using facial expressions gradually deteriorated. He was still able to flex his left toe to control the communication device, but his energy appeared to diminish when attempting to focus on communication. He did not have muscle cramps, and his diabetes mellitus was well controlled with 25mg of alogliptin without renal insufficiency. His medical history included a colonic polyp, which was resected via endoscopy four years previously, and pneumothorax, which was treated conservatively one year previously. He had no history of rhabdomyolysis.

Laboratory evaluations performed to determine the cause of his altered mental state showed that his electrolytes, hematological parameters, including HbA1c, and liver and renal function parameters were within normal ranges (Table 1). However, his serum total carnitine was abnormally low, at 26.7μmol/L (normal range, 45 to 91μmol/L), as were his free carnitine (20.6μmol/L; normal range, 36 to 74μmol/L) and acylcarnitine ester (6.1μmol/L; normal range, 6 to 23μmol/L) levels. However, his ammonium level was normal (48μg/dL; normal range, 12 to 68μg/dL).

**Table 1:** Laboratory tests (29 June 2012)

**Parameter**	**Laboratory result**	**Parameter**	**Laboratory result**
White blood cells	5800/μL	Total cholesterol	157mg/dL
Red blood cells	419 × 10^4^/μL	Triglyceride	165mg/dL
Hemoglobin	12.3g/dL	High density lipoprotein cholesterol	33mg/dL
Platelets	20.4 × 10^4^/μL	Low density lipoprotein cholesterol	100mg/dL
Total protein	7.2g/dL	Sodium	142mEq/L
Creatinine kinase	22U/L	Potassium	3.7mEq/L
Total bilirubin	0.3mg/dL	Chloride	106mEq/L
Aspartate transaminase	17U/L	Blood urea nitrogen	21.9mg/dL
Alanine transaminase	10U/L	Creatinine	0.14mg/dL
Lactate dehydrogenase	145U/L	Uric acid	5.8mg/dL
Gamma-glutamyl transpeptidase	26U/L	Blood glucose	192mg/dL
Cholinesterase	237U/L	HbA1c^a^	5.8%
Amylase	101U/L			

^a^National Glycohemoglobin Standardization Program.

Within two weeks of starting carnitine supplementation (600mg twice daily via the PEG tube), our patient’s mental state improved to the level noted the previous year. His serum total carnitine recovered to 172.3μmol/L (normal range, 45 to 91μmol/L) one year after starting carnitine supplementation. His free carnitine (140.6μmol/L; normal range, 36 to 74μmol/L) and acylcarnitine ester (31.7μmol/L; normal range, 6 to 23μmol/L) levels had also recovered at this time. Our patient’s mental state has been maintained since starting carnitine supplementation.

## Discussion

L-carnitine is found in a variety of food sources but is highly abundant in animal products. Ensure liquid® is made from soybeans and casein, which are low in carnitine [5]. Our patient was given coffee with milk, but his total dietary carnitine intake appeared to be low. In healthy individuals, low carnitine intake does not always lead to carnitine deficiency because of abundant muscle stores, efficient reabsorption in the kidneys, and endogenous synthesis in the liver from lysine and methionine, which are present in sufficient amounts in Ensure liquid®. Normally, more than 95% of the body’s total carnitine content would be stored in skeletal muscle [6,7]. However, in patients with ALS, severe muscle atrophy could reduce the carnitine stores, thus contributing to secondary carnitine deficiency caused by long-term enteral feeding with low-carnitine products.

Decreased plasma carnitine levels are an early marker of impending secondary carnitine deficiency in tissues. Low carnitine levels result in the accumulation of unoxidized fatty acids, causing hyperammonemia through inhibition of the urea cycle. Indeed, carnitine deficiency has been reported in patients with hyperammonemia [8,9]. However, hyperammonemia was not seen in our patient. Anticonvulsants such as phenytoin and valproate [5,8] and long-term administration of pivalate-containing antibiotics [10] have been reported to lower carnitine levels. Our patient did not take any drugs known to reduce carnitine levels.

Measuring plasma carnitine levels may help clinicians to differentiate between the progression of ALS and carnitine deficiency in patients with gradual impairments in communication, especially in patients receiving long-term tube feeding with commercial products. In transgenic mouse models with similar phenotypes to ALS in humans, muscle apoptosis was prevented by L-carnitine supplementation [11]. However, further research on L-carnitine supplementation is needed to confirm its role in preventing disease progression in patients with ALS.

## Conclusions

Patients with ALS experience progressive muscle atrophy; therefore, it is inevitable that they will lose the ability to communicate over time. Many Japanese patients with ALS requiring PEG feeding are given nutritional products approved by the Ministry of Health, Labor and Welfare that are made from soybeans and casein, but frequently contain low levels of L-carnitine. To avoid gradual impairments in communication caused by secondary carnitine deficiency, clinicians should be aware of and regularly measure their patients’ carnitine levels, regardless of their serum ammonia levels. If carnitine supplementation is necessary, it should be administered in the form of L-carnitine tablets or other products containing carnitine.

## Consent

Written informed consent was obtained from the patient and his family for the publication of this case report. A copy of the written consent is available for review by the Editor-in-Chief of this journal.

## Abbreviations

ALS: Amyotrophic lateral sclerosis; PEG: Percutaneous endoscopic gastrostomy.

## Competing interests

The authors declare that they have no competing interests.

## Authors’ contributions

TO and NT analyzed and interpreted the patient’s data regarding carnitine deficiency. YM was primarily responsible for the patient. NI was the lead writer of this manuscript. All authors read and approved the final manuscript.

## References

[B1] Rebouche CJ Paulson DJ Carnitine metabolism and function in humans Ann Rev Nutr 1986 6 41 66 10.1146/annurev.nu.06.070186.000353 3524622

[B2] Ling P Lee DJ Yoshida EM Sirrs S Carnitine deficiency presenting with encephalopathy and hyperammonemia in a patient receiving chronic enteral tube feeding: a case report J Med Case Rep 2012 6 227 10.1186/1752-1947-6-227 22846666 PMC3432593

[B3] Brass EP Adler S Sietsema KE Hiatt WR Orlando AM Amato A Intravenous L-carnitine increases plasma carnitine, reduces fatigue, and may preserve exercise capacity in hemodialysis patients Am J Kidney Dis 2001 37 5 1018 1028 10.1016/S0272-6386(05)80019-8 11325685

[B4] Cruciani RA Zhang JJ Manola J Cella D Ansari B Fisch MJ L-carnitine supplementation for the management of fatigue in patients with cancer: an eastern cooperative oncology group phase Ш, randomized, double-blind, placebo-controlled trial J Clini Oncol 2012 30 31 3864 3869 10.1200/JCO.2011.40.2180 PMC3478577 22987089

[B5] Tanaka S Miki T Hsieh ST Kim JI Yasumoto T Taniguchi T Ishikawa Y Yokoyama M A case of severe hyperlipidemia caused by long-term tube feedings J Atheroscler Thromb 2003 10 5 321 324 10.5551/jat.10.321 14718750

[B6] Maeda K Nakai S Carnitine deficiency and nutritional measures Jpn J Clin Dial 2001 17 12 1597 1602

[B7] Flanagan JL Simmons PA Vehige J Willcox MD Garrett Q Role of carnitine in disease Nutr Metab 2010 7 30 10.1186/1743-7075-7-30 PMC2861661 20398344

[B8] Ohtani Y Endo F Matsuda I Carnitine deficiency and hyperammonemia associated with valproic acid therapy J Pediatr 1982 101 5 782 785 10.1016/S0022-3476(82)80320-X 6813444

[B9] Limketkai BN Zucker SD Hyperammonemic encephalopathy caused by carnitine deficiency J Gen Intern Med 2008 23 210 213 10.1007/s11606-007-0473-0 18080167 PMC2359173

[B10] Nakajima Y Ito T Maeda Y Ichiki S Sugiyama N Mizuno M Makino Y Sugiura T Kurono Y Togari H Detection of pivaloylcarnitine in pediatric patients with hypocarnitinemia after long-term administration of pivalate-containing antibiotics Tohoku J Exp Med 2010 221 309 313 10.1620/tjem.221.309 20651467

[B11] Patel BP Hamadeh MJ Nutritional and exercise-based interventions in the treatment of amyotrophic lateral sclerosis Clin Nutr 2009 28 6 604 617 10.1016/j.clnu.2009.06.002 19782443

